# Discovery of a Rare Pterosaur Bone Bed in a Cretaceous Desert with Insights on Ontogeny and Behavior of Flying Reptiles

**DOI:** 10.1371/journal.pone.0100005

**Published:** 2014-08-13

**Authors:** Paulo C. Manzig, Alexander W. A. Kellner, Luiz C. Weinschütz, Carlos E. Fragoso, Cristina S. Vega, Gilson B. Guimarães, Luiz C. Godoy, Antonio Liccardo, João H. Z. Ricetti, Camila C. de Moura

**Affiliations:** 1 Centro Paleontológico da UnC (CENPÁLEO), Universidade do Contestado, Mafra, Santa Catarina, Brazil; 2 Programa de Pós-Graduação IEL-Labjor, Universidade Estadual de Campinas (UNICAMP), Campinas, São Paulo, Brazil; 3 Laboratory of Systematics and Taphonomy of Fossil Vertebrates, Departamento de Geologia e Paleontologia, Museu Nacional/Universidade Federal do Rio de Janeiro, Rio de Janeiro, Brazil; 4 Universidade Estadual de Ponta Grossa, Ponta Grossa, Paraná, Brazil; 5 Departamento de Geologia, Universidade Federal do Paraná, Curitiba, Paraná, Brazil; 6 Departamento de Geociências, Universidade Estadual de Ponta Grossa, Ponta Grossa, Paraná, Brazil; Raymond M. Alf Museum of Paleontology, United States of America

## Abstract

A pterosaur bone bed with at least 47 individuals (wing spans: 0.65–2.35 m) of a new species is reported from southern Brazil from an interdunal lake deposit of a Cretaceous desert, shedding new light on several biological aspects of those flying reptiles. The material represents a new pterosaur, *Caiuajara dobruskii* gen. et sp. nov., that is the southermost occurrence of the edentulous clade Tapejaridae (Tapejarinae, Pterodactyloidea) recovered so far. *Caiuajara dobruskii* differs from all other members of this clade in several cranial features, including the presence of a ventral sagittal bony expansion projected inside the nasoantorbital fenestra, which is formed by the premaxillae; and features of the lower jaw, like a marked rounded depression in the occlusal concavity of the dentary. Ontogenetic variation of *Caiuajara dobruskii* is mainly reflected in the size and inclination of the premaxillary crest, changing from small and inclined (∼115°) in juveniles to large and steep (∼90°) in adults. No particular ontogenetic features are observed in postcranial elements. The available information suggests that this species was gregarious, living in colonies, and most likely precocial, being able to fly at a very young age, which might have been a general trend for at least derived pterosaurs.

## Introduction

Pterosaurs comprise an extinct group of flying reptiles that have been recovered on all continents [1]. Notwithstanding their distribution, their record is rather patchy, with most occurrences limited to fragmentary remains that in several cases were only briefly reported in the literature [2]. Most pterosaurs are known from ancient coastal or shallow marine deposits and the number of species that lived deep inside the continents is limited [3], [4], particularly from desert environments [5]. Most species are based on one incomplete individual, and aside from one potential exception of a collection of flattened specimens [6], no pterosaur accumulation can be regarded as a bone bed preserving several individuals that can confidently be assigned to the same species and at least potentially be regarded as representing the same or successive populations [7]. This has hampered the discussion of several biological questions regarding those animals, such as ontogenetic growth, development of cranial crests, and behavior.

**Figure 1:**
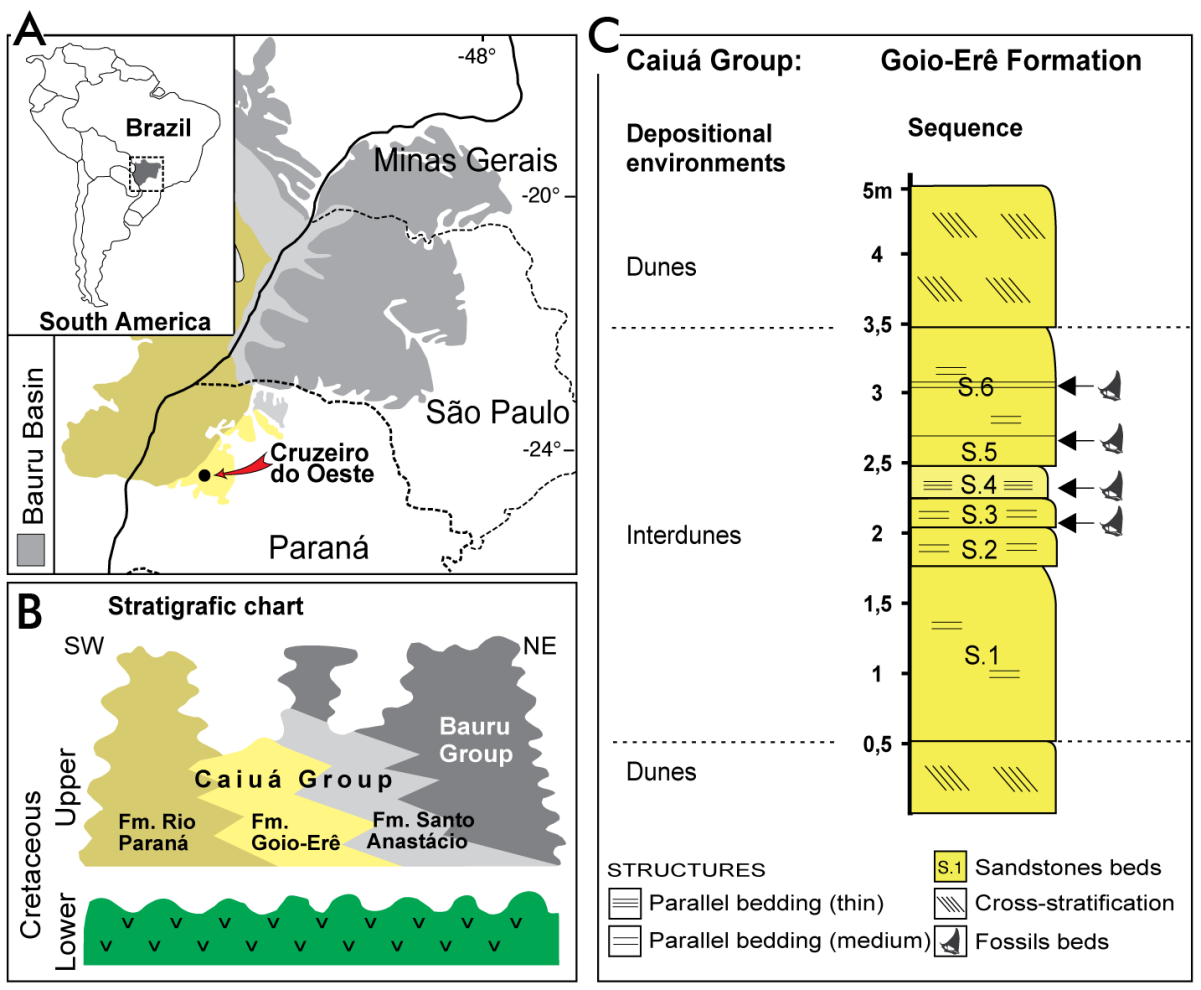
Localization and stratigraphic framework of the new pterosaur locality. (**A**) Map of South America and the geographic position of Cruzeiro do Oeste. (**B**) Stratigraphic chart showing the relation between the distinct stratigraphic units of the Bauru Basin [10]. (**C**) Detailed stratigraphic section of the quarried beds of the Goio-Erê Formation, showing the location where the fossils were recovered.

Here we describe a rare pterosaur bone bed composed of hundreds of bones from the outskirts of Cruzeiro do Oeste, southern Brazil. The deposits correspond to the Caiuá Group [8] that represents a sand sea formed in an interior paleodesert whose paleontological content was up to know limited to infrequent tetrapod ichnofossils [9], [10] (Figure 1). This exceptional occurrence, combined with the large number of three-dimensionally preserved individuals, sheds new light on the biology of those rather enigmatic volant animals.

## Materials and Methods

### Phylogenetic Analysis

In order to determine the phylogenetic position of *Caiuajara dobruskii* gen. et sp. nov., we performed a phylogenetic analysis using PAUP 4.0b10 for Microsoft Windows [11] using the TBR heuristic searches performed using maximum parsimony. Characters were given equal weight and treated as unordered (ACCTRAN setting). This analysis is based on previous cladistic studies (List S3 in File S1).

### Nomenclatural Acts

The electronic edition of this article conforms to the requirements of the amended International Code of Zoological Nomenclature, and hence the new names contained herein are available under the Code from the electronic edition of this article. This published work and the nomenclatural acts it contains have been registered in ZooBank, the online registration system for the ICZN. The ZooBank LSIDs (Life Science Identifiers) can be resolved and the associated information viewed through any standard web browser by appending the LSID to the previx “http://zoobank.org/”. The LSID for this publication is: urn:lsid:zoobank.org:pub:E6A57D0A-3F3A-4F56-9279-B12CFA222337. The electronic edition of this work was published in a journal with an ISSN, and has been archieved and is available from the following digital repositories: PubMed Central, LOCKSS.

All permits were obtained for the described study, which complied with all relevant regulations. The permit for collecting the specimens was issued by the Departamento Nacional de Produção Mineral (DNPM, Brasília), under the number DNPM n° 48400-000807/2012-94. See appropriate section of Systematic Paleontology for locality, stratigraphy and repository, and specimen numbers.

## Results

### Systematic Paleontology

**Pterosauria** Kaup, 1834

**Pterodactyloidea** Plieninger, 1901

**Azhdarchoidea** Nessov, 1984

**Tapejaridae** Kellner, 1989

**Tapejarinae** Kellner, 1989 *sensu* Kellner & Campos [12]

***Caiuajara dobruskii*** gen. et sp. nov.

**ZooBank Life Science Identifier (LSID) for genus.**

urn:lsid:zoobank.org:act:9E5919F7-7A2A-4065-9FC1-11EB1960BF5C

**ZooBank LSID for species.** urn:lsid:zoobank.org:act:CF251616-A7AA-4C25-A6BE-B69AB448D93B

#### Etymology

Combination of Caiuá and *Tapejara*, the internal specifier of the Tapejarinae [13]; species honors Alexandre Dobruski, who with his son, João Dobruski, found the new site back in 1971.

#### Holotype

**Figure 2:**
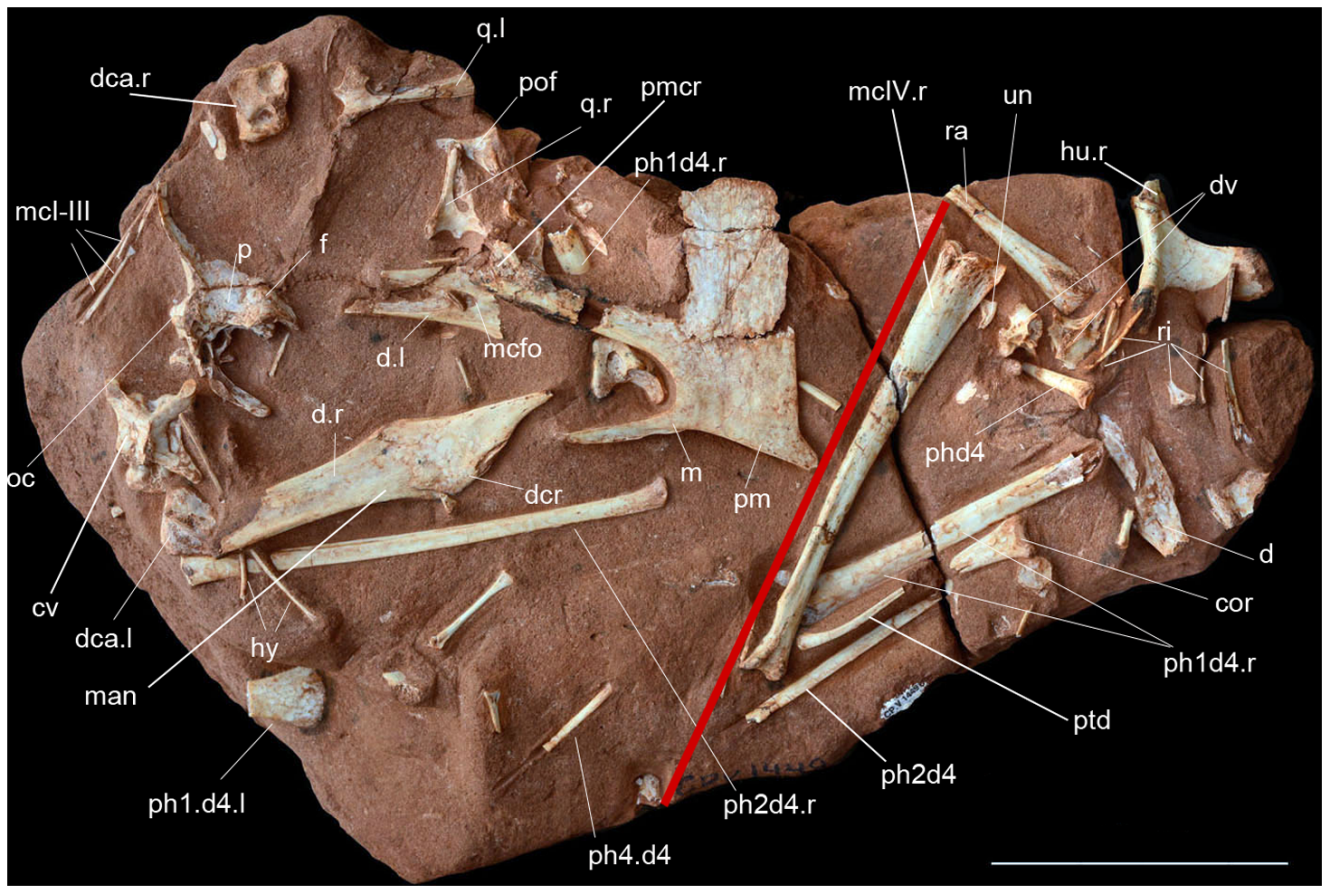
Holotype (CP.V 1449 left) and one paratype (CP.V 2003, right) of *Caiuajara dobruskii* gen. et sp. nov. separated by a red line, showing skull and postcranial elements. Scale bar equals 100: cor, coracoid; cv, cervical vertebra; d, dentary; dca, distal carpal series; dcr, dentary crest; f, frontal; hu, humerus; hy, hyoid bone; l, left; man, mandible; mcfo, meckelian fossa; mcI-III, metacarpal I–III; mcIV, metacarpal IV; oc, occipital condyle; p, parietal; pmcr, premaxillary crest; ph1d4, first phalanx of manual digit IV; ph2d4, second phalanx of manual digit IV; ph4d4, forth phalanx of manual digit IV; pof, postfrontal; ptd, pteroid; q, quadrate; r, right; ra, radius; ri, rib; un, ungueal.

**Figure 3:**
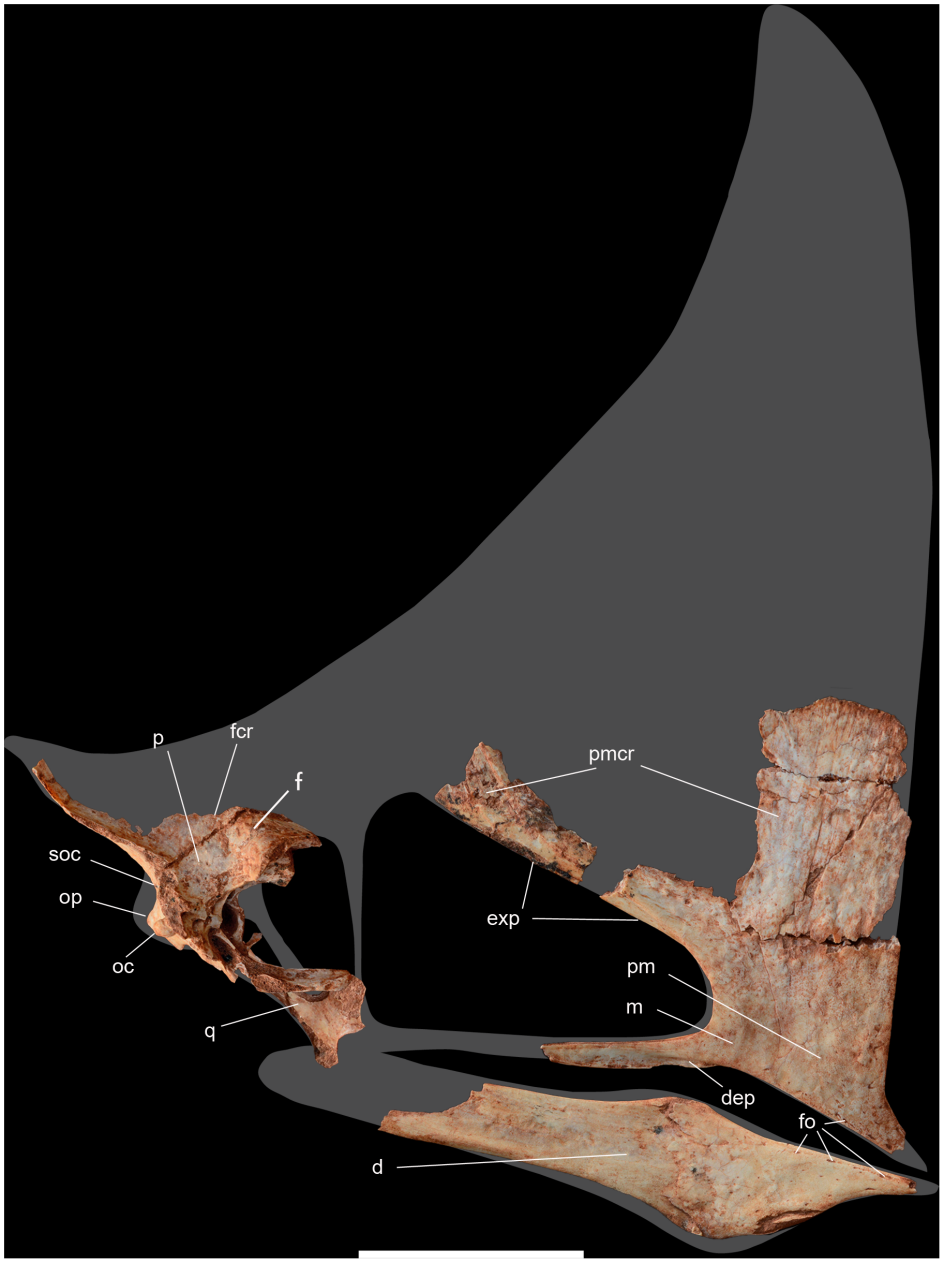
Skull of *Caiuajara dobruskii* gen. et sp. nov. (holotype, CP.V 1449) with the shape of an adult individual. Scale bar equals 50: d, dentary; dcr, dentary crest; dep, depression; exp, ventral expansion of the premaxilla; f, frontal; fcr, frontal crest; fo, foraminae; m, maxilla; oc, occipital condyle; op, opisthotic; p, parietal; pm, premaxilla; pmcr, premaxillary crest; q, quadrate; soc, supraoccipital. The quadrate is inverted.

Partial skeleton including skull and lower jaw, cervical vertebrae and wing elements (CP.V 1449), housed at the Centro Paleontológico (CENPALEO) of the Universidade do Contestado, Mafra, Santa Catarina, Brazil (Figures 2, 3).

#### Paratypes

**Figure 4:**
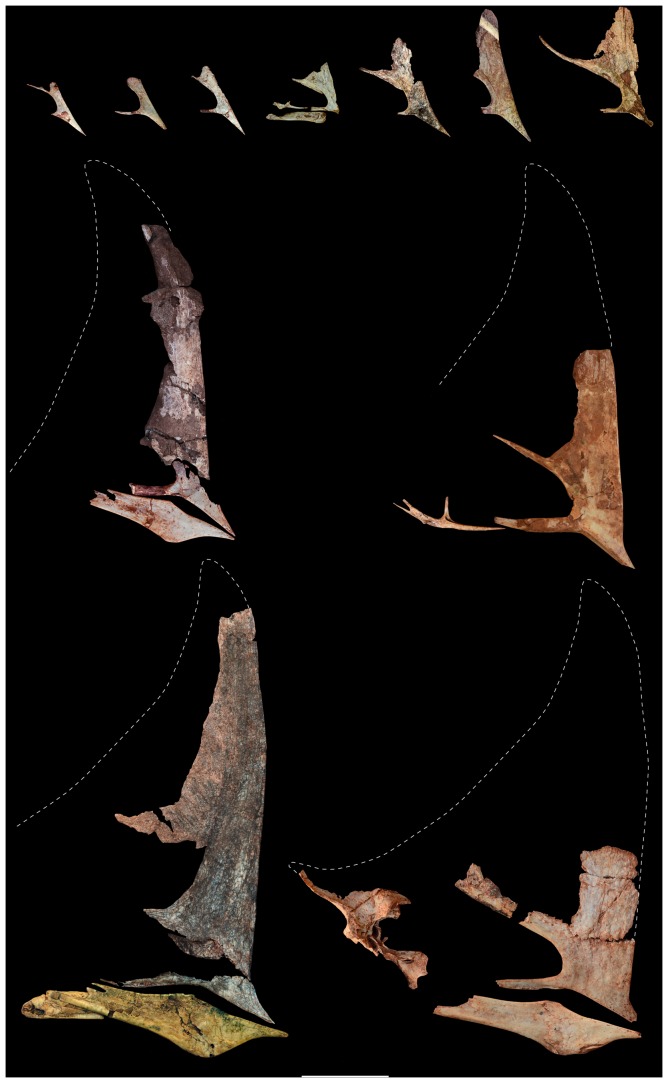
Selected cranial material of *Caiuajara dobruskii* showing anatomical changes during ontogeny. Note that the cranial crest gets gradually larger in older individuals. From top left to bottom right: CP.V 1050-1 (inverted), CP.V 1050-2 (inverted), CP.V 1003, CP.V 866 (inverted), UEPG/DEGEO/MP-4151, CP.V 1023 (inverted), UEPG/DEGEO/MP-4151 (second skull), CP.V 1001, CP.V 1447, CP.V 1005 (with posterior part of lower jaw reconstructed), CP.V 1449 (holotype). Scale bar, equals 50 mm.

**Figure 5:**
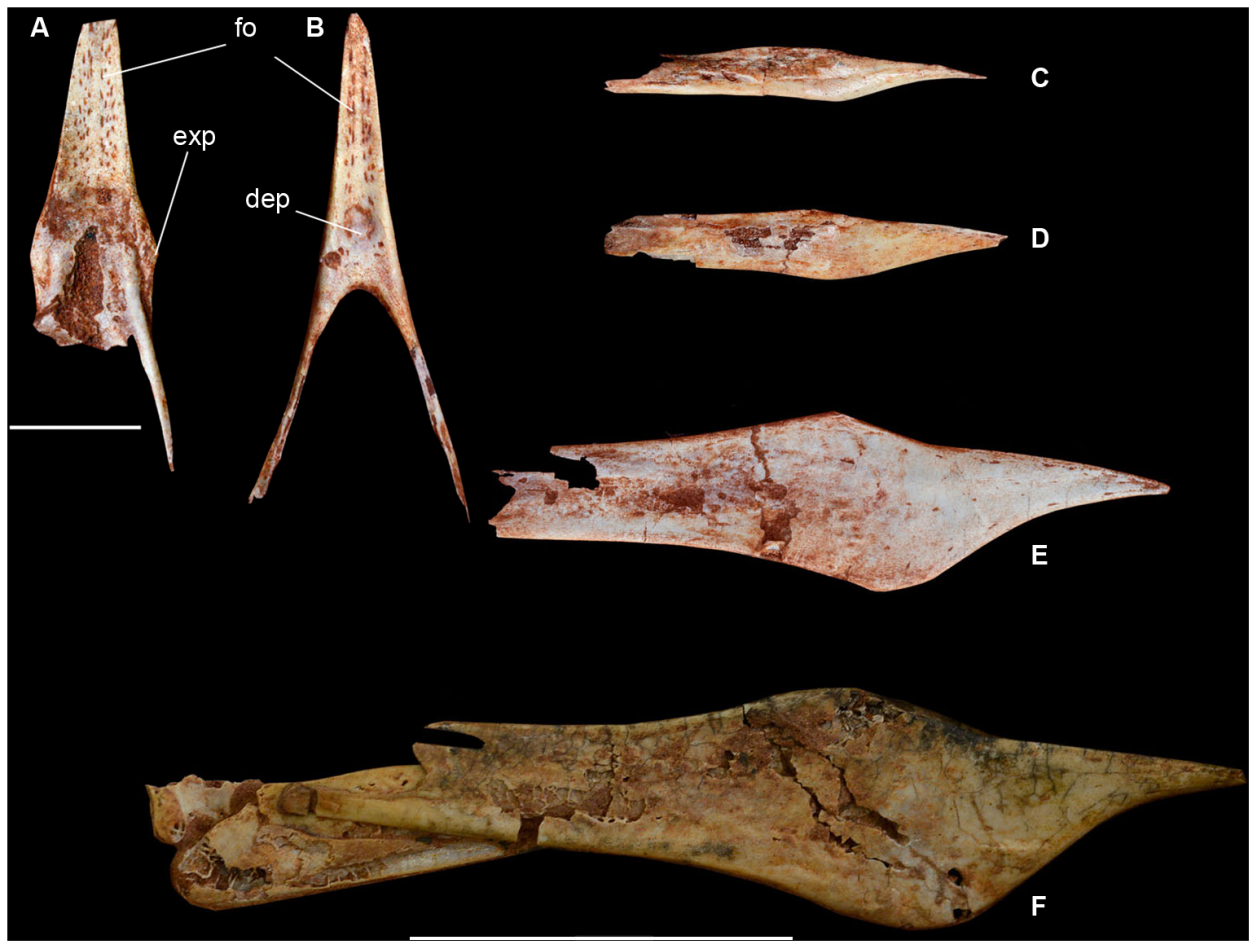
*Caiuajara dobruskii* gen. et sp. nov., occlusal view of upper jaw (A) and mandible (B) (CP.V 1449). Scale bar equals 10; different lower jaws, from from top to bottom: juvenile (CP.V 1450-2) (C); older juvenile (CP.V. 1450-1) (D); young/subadult (CP.V. 1001a-1) (E); and adult (CP.V 1005a) (F) specimen. Scale bar equals 50 mm. Abbreviations: dep, depression; exp, lateral expansion; fo, foraminae.

**Figure 6:**
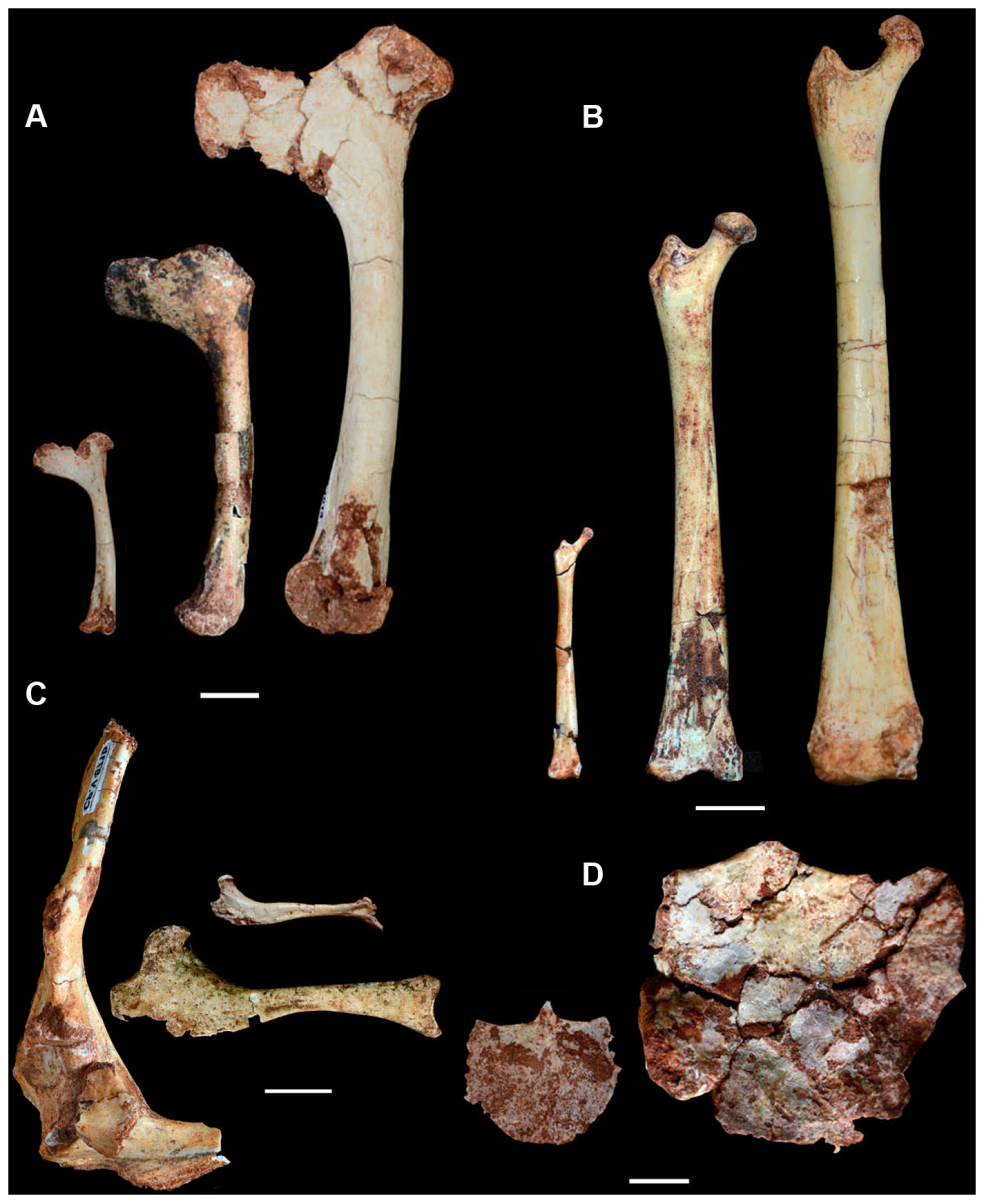
Selected post-cranial elements of *Caiuajara dobruskii* showing the anatomical changes during ontogeny. A, humeri (CP.V 1450 - inverted; CP.V 1009; CP.V 1013); B, femura (CP.V 1883-1; CPV 872a-1; CP.V 1025), scapulocoracoid (CP.V 871b - inverted), coracoids (CP.V 1006-1; CP.V 866b - inverted); and sterna (CP.V 1000; CP.V 1001a-1). Scale bar equals 10 mm.

**Figure 7:**
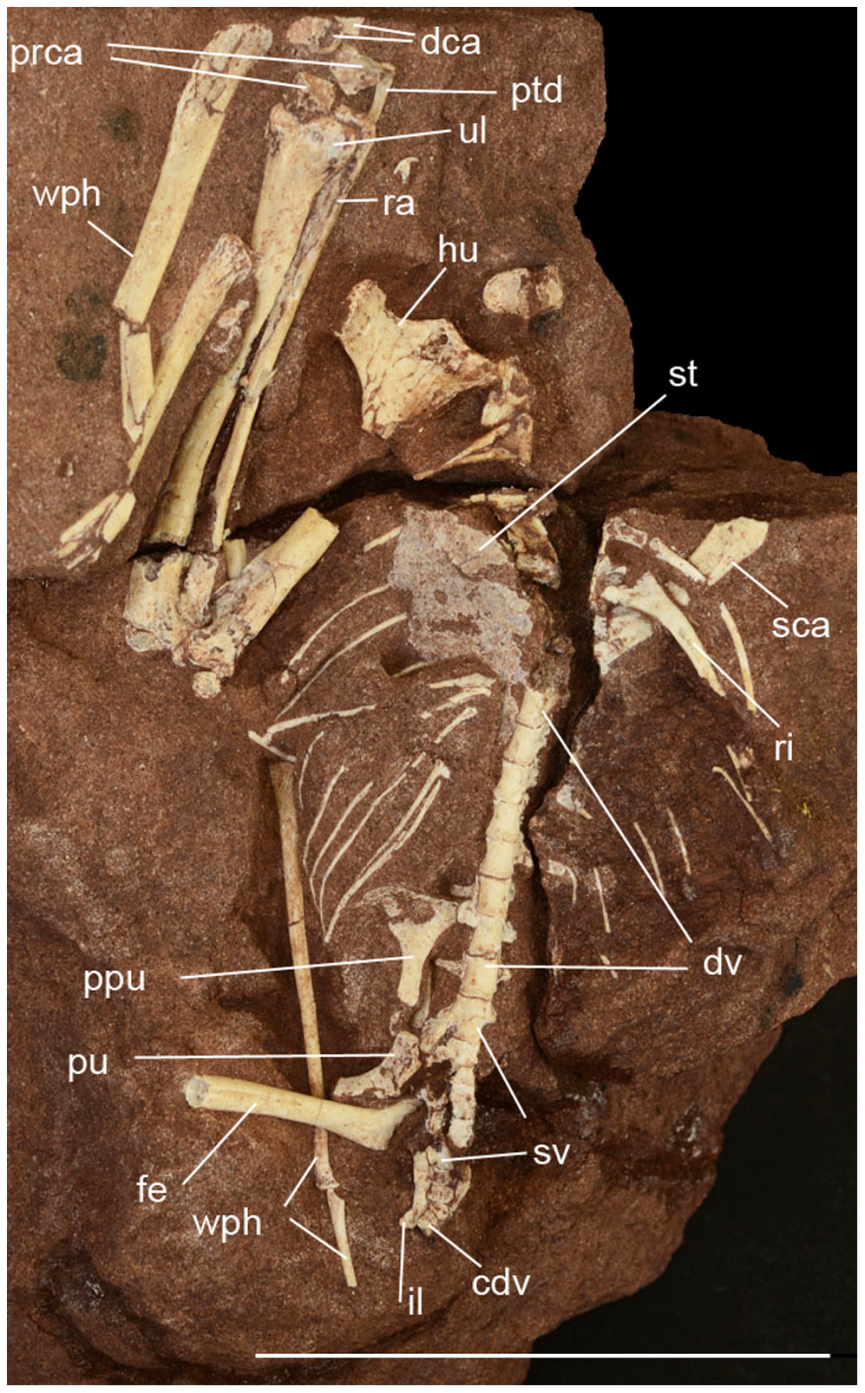
*Caiuajara dobruskii* gen. et sp. nov., (CP.V 869), partial articulated skeleton. Scale bar equals 50: cdv, caudal vertebrae; dca, distal carpal series; dv, dorsal vertebrae; fe, femur; hu, humerus; il, ilium; prca, proximal carpal series; ptd, pteroid; ppu, prepubis; pu, pubis; ra, radius; ri, ribs; sca, scapula; st, sternum; sv, sacral vertebrae; ul, ulna; wph, wing phalanx.

**Figure 8:**
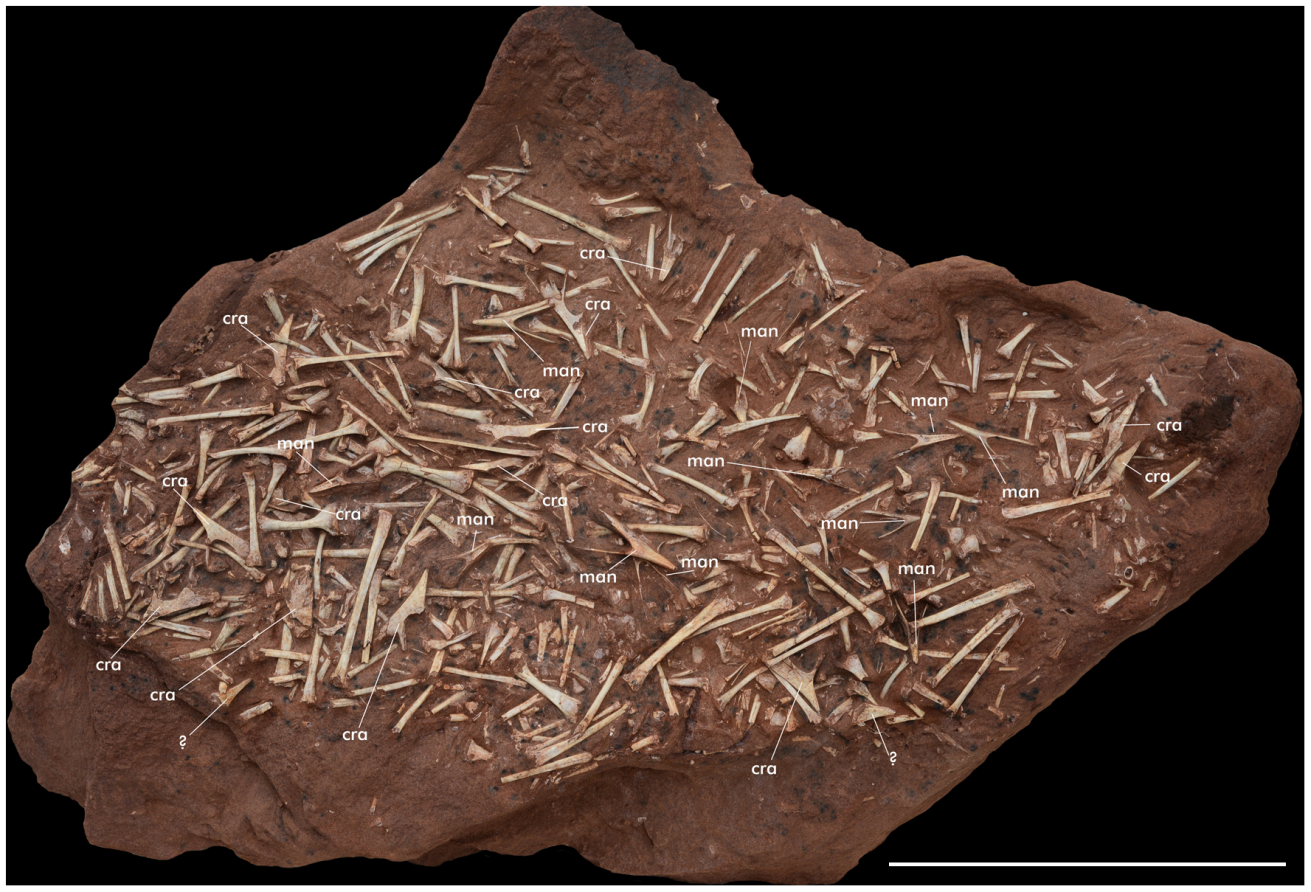
Hundreds of bones, including at least 14 partial skulls of *Caiuajara dobruskii* (CP.V. 1450). Scale bar equals 200: cra - skulls, man - mandible.

CP.V 865, consisting of the anterior portion of a skull, the posterior portion of the lower jaw, the right jugal, vertebrae, ribs and metatarsals; CP.V 867, rostral end of a skull and long bones; CP.V 868, rostral end of a skull, wing elements and other postcranial bones; CP.V 869, incomplete skeleton with a partial vertebral column (posterior cervicals vertebrae, dorsal elements to the first five caudal vertebrae), right humerus, radius and ulna, carpal elements, coracoid, sternum, some wing phalanges, gastralia, pelvic elements and the right femur; CP.V 870, incomplete postcranial elements, with humeri and pectoral girdle; CP.V 871, fused right scapulocoracoid and incomplete long bones; CP.V 872, partial skeleton including a fragmentary skull, lower jaw, right humerus, radius, ulna, carpals, cervical vertebrae and other long bones; CP.V 873, rostral end of a skull and manual phalanges; CP.V 999, partial skull; CP.V 1001, one incomplete skull with elongated premaxillary crest and lower jaw, and postcranial elements of at least three individuals, wing bones (with humeri), cervical vertebrae and pelvic elements; CP.V 1003, incomplete skull and the rostral tip of the lower jaw; CP.V 1004, rostral end of a skull; CP.V 1005, incomplete skull with an elongated premaxillary crest and a complete lower jaw; CP.V 1006, partial skull with anterior rostral end missing, with large premaxillary crest and several postcranial bones; CP.V 1023, anterior portion of a skull and several postcranial elements; CP.V 1024, skull and several postcranial bones of at least three small individuals; CP.V 1025, isolated femur; CP.V 1026, isolated femur; CP.V 1450, several small individuals (at least 14); CP.V 2003, skull and lower jaw associated with wing bones; UEPG/DEGEO/MP-4151, two skulls on one slab and postcranial elements; and UEPG/DEGEO/MP-4152, a rostrum and several postcranial elements (Figures 2, 4–8). For referred specimens, see List S1 in File S1.

#### Type locality, horizon and age

Cruzeiro do Oeste, Paraná State, Brazil; Bauru Basin, Caiuá Group, Goio-Erê Formation, Upper Cretaceous [8], [12], [14].

#### Diagnosis

Tapejarine tapejarid with the following autapomorphies: anterior end of the premaxillary strongly deflected ventrally (∼142–149°) relative to the ventral margin of the upper jaw; premaxillae with ventral sagittal bony expansion projected inside the nasoantorbital fenestra; rounded depression in the occlusal concavity of the dentary; elongates groove on the anterolateral margin of the quadrate; and marked lateral depression on maxilla ventral to anterior part of the nasoantorbital fenestra. The new species can be further distinguished from other tapejarine pterosaurs by the following combination of characters: ventral margin of the orbit rounded; gap between upper and lower jaws during occlusion wider; and marked depression on ventral side of the pteroid lacking a pneumatic foramen.

### Description and Comparisons

Several anatomical features show that *Caiuajara* belongs to the toothless pterodactyloid clade Tapejaridae [13], such as a premaxillary crest from the anterior rostral end extended above the occipital region, nasoantorbital fenestra elongated comprising more than 40% of the cranial length (Figure 3), and a well-developed tubercle on anterior surface of the coracoid. It further has all synapomorphies of the Tapejarinae, such as the downturned anterior part of the rostrum [12], [13], [15]–[17], with the inclination varying from about 138° to 150°; most species average 142°. The orbit is piriform, with the ventral margin more rounded than in other tapejarids [15], [18]. The nasoantorbital fenestra anterior margin is wide, similar to other tapejarines [15], [16], [19] but differing from the narrower condition of thalassodromines [12], [20].

The premaxilla is perforated by a large number of foraminae on the lateral and palatal surface, similar to *Tapejara*, suggesting that the beak was covered by a horny covering analogous to the rhamphoteca in birds. A developed premaxillary sagittal crest is present in the smallest and the largest individuals (Figure 4), casting doubts on previous interpretations that the presence and absence of cranial crests might be sexually dimorphic [21]. The anterior part of the crest is very high similar to *Tupandactylus* [13], [22], but differs by being more expanded. The occipital portion of the crest, formed by the supraoccipital and parietal, is dorsally curved, differing from the much longer and straighter structure found in *Tupandactylus* [22]. Starting close to the anterior margin of the nasoantorbital fenestra, the premaxilla has a sagittal bony expansion that extends posteriorly, where it merges with the lateral margin of this opening (Figure 3). This structure, whose function is unknown, is present in all specimens, from the smallest to the largest, and has not been reported in any other pterosaur before (Figure 4). As in *Tapejara*, *Caiuajara* shows a deep concavity in the palate restricted to the anterior part, followed by a posterior convexity. Differing from all other tapejarids where the occlusal surface can be observed, some specimens of *Caiuajara* have a faint longitudinal crest inside the most concave portion of the palate that does not form a palatal ridge as in some thalassodromines [12] and some pteranodontoids [17], [23]. As in other tapejarines, the upper jaw shows a small lateral expansion close to the anterior margin of the nasoantorbital fenestra. The occlusal surface of the dentary also displays a deep concavity as in *Tapejara*, but *Caiuajara* differs by showing a distinctive rounded depression (Figure 5). The dentary shows the typical tapejarine step-like dorsal margin and a blunt dentary sagittal crest that is more developed in larger individuals, similar to *Tapejara* [18] and *Europejara* [24], but differing from the Chinese tapejarines [15], [16]. There is no helical jaw joint, differing from *Caupedactylus* [25].

The cervical vertebrae (Figure 2) are slightly elongated, more so than in pteranodontoids [26], but not to the same degree as in archaeopterodactyloids [17], [23] or azhdarchids [27], [28]. The neural spine is blade-like and the centrum pierced laterally by small pneumatic foraminae. One lateral pneumatic opening occurs on each side of the neural canal on the anterior articulation surface. No notarium is developed. The sacrum is formed by five sacrals. The scapula is longer than coracoid, and where complete, the coracoid shows a developed tubercle on the anterior surface (Figure 6). The sternum is semicircular in shape. The humerus displays an elongated deltopectoral crest that is rectangular and slightly curved medially, particularly at the most posterodistal end, but the crest is not warped as in pteranodontoids [17], [23], [26], [29]. About 35 humeri were identified so far (20 right, 14 left and one unidentifiable) with lengths ranging from 31 mm to 115.6 mm. Overall, the radius is thinner than the ulna, but not to the same degree as observed in istiodactylids and anhanguerids [23]. Distal sincarpals show a rectangular shape. The pteroid clearly articulates with the proximal carpal series, showing a developed ventral depression but no pneumatic foramen (Figure 7). Wing metacarpal IV is similar to that seen in other tapejarids, being proportionally longer relative to other wing elements when compared to anhanguerids (Figure 2), but does not approach the extreme elongation reported in nyctosaurids [23], [26]. The femur is bowed and about the same size as the humerus (Figure 6).

**Figure 9:**
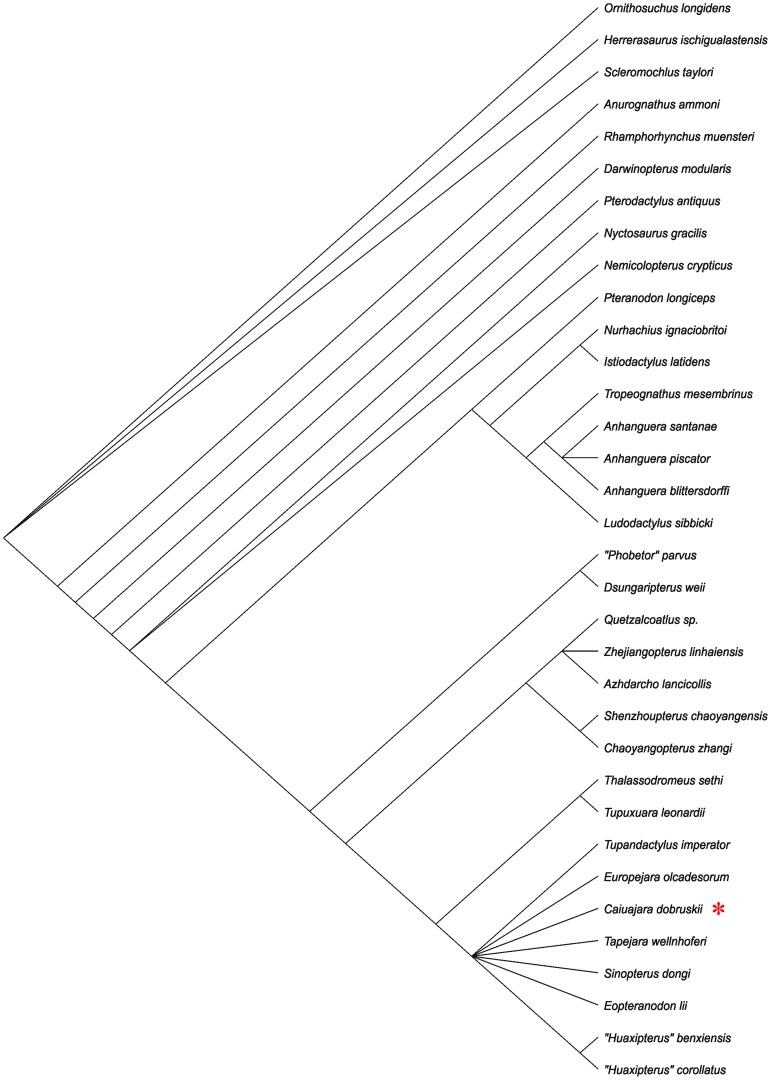
Phylogenetic analysis showing the relationships of *Caiuajara dobruskii* gen. et sp. nov. Based on Vullo et al. 2012 [23].

A phylogenetic analysis based on a previous study of tapejarid phylogenetic relationships [24] shows that *Caiuajara* is a member of the Tapejarinae, falling in a polytomy with other tapejarine tapejarids (Figure 9). If *Eopteranodon*, a poorly described taxon, and *Europejara* (unfortuntately very incomplete) are removed, *Cauiajara* falls in a sister group relationship with *Tupandactylus*, in a trichotomy with *Tapejara* and *Sinopterus*+*Huaxiapterus*, indicating that the known tapejarines from China form a monophyletic entity. This exercise also shows that much more has to be done to resolve the relationships of the Tapejaridae, particularly the Tapejarinae.

Previous studies of pterosaur ontogeny were based on isolated specimens mostly recovered without stratigraphic control that, despite important contributions [30]–[39], have fostered some controversy, particularly over whether or not the studied specimens represent the same species [40], [41]. *Caiuajara* is the first case where a pterosaur ontogenetic series is provided based on specimens from a pterosaur bone bed that can be confidently assigned to the same species. The sample also has the advantage of having most elements preserved three-dimensionally and not flattened, avoiding the problems related to change of morphology due to distortion [6], [7], [36]. Regarding postcranial elements, there are few differences from smaller to larger individuals except for size and the tendency for ontogenetically more developed individuals to show more ossified bones, particularly the sternum (Figure 6). The humerus, for example, shows the same proportion in smaller and larger individuals, including the development of the deltopectoral crest that corresponds to about 38–40% of the humerus length. This indicates that the general shape of most postcranial elements is formed at a juvenile stage and does not change significantly, as the animal grows older. The most conspicuous exceptions are the prepubis, with older individuals showing a more developed and larger distal plate, and the coracoid, where ontogenetically more developed individuals display a slightly larger ventral expansion. Furthermore, as reported in other pterodactyloids, the scapula and coracoid are fused in adult individuals but unfused in younger individuals, with the same trend happening in the epiphyses of the humerus and the carpal series [32], [35].

**Figure 10:**
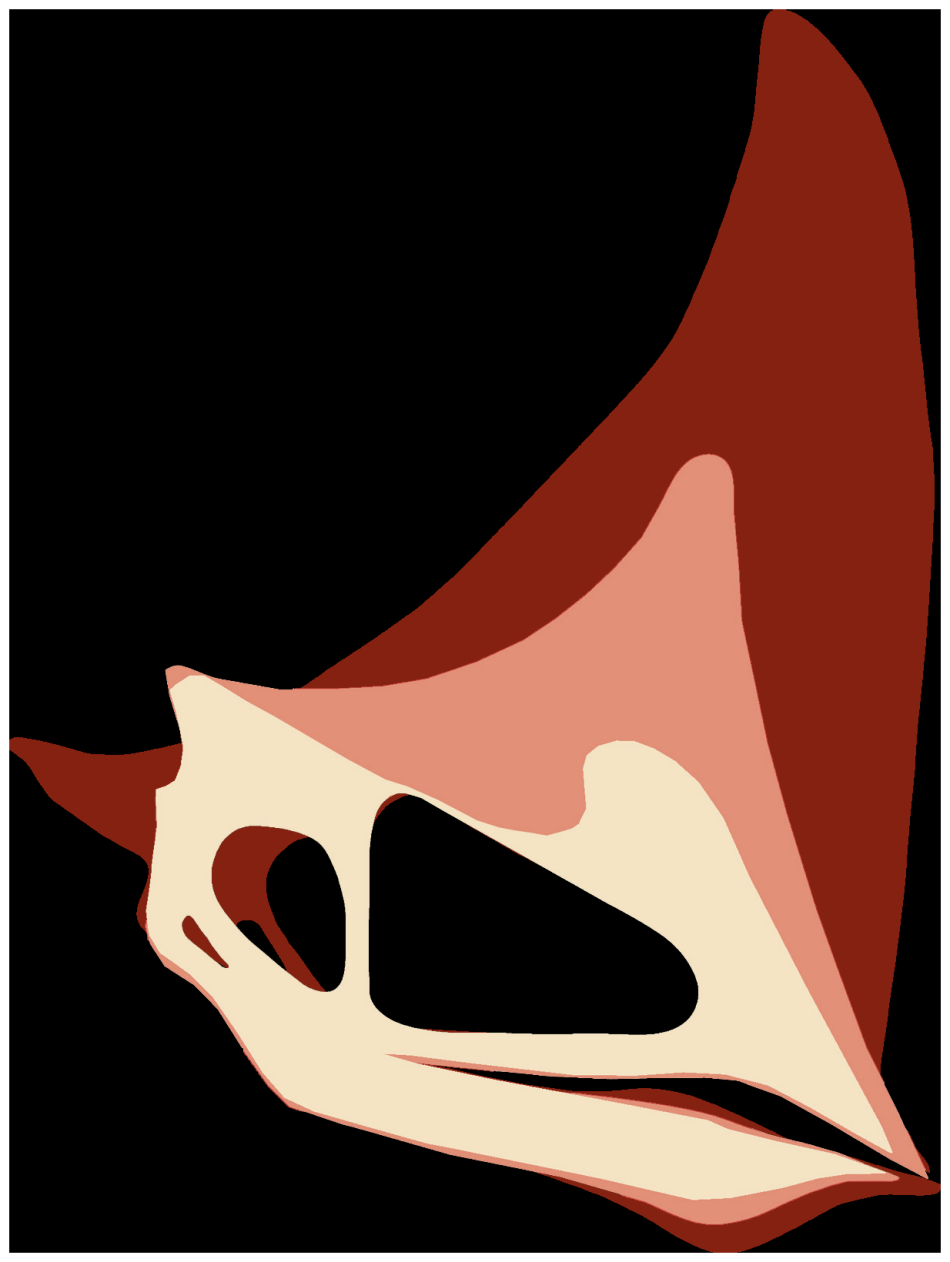
*Caiuajara dobruskii* gen. et sp. nov., reconstruction of shapes from juveniles (bright color) to adults (darker color). Outlines not to scale.

Regarding the skull, the main ontogenetic differences can be found in the rostrum and the cranial crest (Figures 4, 10). Younger individuals display a reduced rostrum that grows, becoming more massive in older individuals. The inclination of the occlusal margin relative to the horizontal plane does not vary significantly, mostly being around 142°. The premaxillary crest, on the contrary, shows marked variation, being reduced and inclined posteriorly for about ∼115° relative to the horizontal plane in small individuals. As the animal grew, the crest got rapidly larger and steeper (up to ∼90°). Similar changes are observed in the dentary crest, which is almost absent in young individuals and gets more developed in older ones (Figures 5, 10).

## Discussion and Conclusions

There are several interesting aspects of this discovery. So far, all other pterosaur material recorded from Brazil comes from the northeastern part of the country [1], and this is the first in the southern part. Besides the Crato and Romualdo formations of Brazil [12], tapejarid pterosaurs have also been recorded in China [15], [16], Morocco [42], and Spain [24], all in deposits that range from the Barremian to the Cenomanian [24]. Based on stratigraphic correlations, the age of the Goio-Erê Formation is regarded as Turonian to Campanian [14], or even having a Coniacian basal limit [10]. Therefore, either *Caiuajara* is the youngest member of this group known to date, or this deposit is older than previously thought. In any case, Cruzeiro do Oeste is the southernmost occurrence of Tapejarinae recorded so far, suggesting that those pterosaurs, which are regarded to be frugivorous [18], [24], had a cosmopolitan distribution.

The discovery of this pterosaur bone bed also allows inference of some aspects regarding the behavior of the new species that might be applied for other flying reptiles. *Caiuajara dobruskii* is known from hundreds of bones representing individuals of different sizes that were collected in an area of less than 20 m^2^. Based on the premaxillae, a minimum of 47 individuals can be established (List S2 in File S1), but the actual number present in this site must be well in the hundreds.

All parts of the skeleton are represented. The skeletons of a few specimens were found articulated (Figure 7) or closely associated (Figure 2), but most are mixed together, making it difficult establish which elements belong to the same individual. In one extreme case, at least 14 individuals could be identified based on the premaxillae in one small block of sandstone (40 cm by 60 cm; more was left in the outcrop) with hundreds of bones, including 11 lower jaws, all belonging to small individuals (Figure 8), indicating that this pterosaur accumulation was partially submitted to hydraulic selection. In several instances, there is indication that bones were broken prior to fossilization and that some might have been exposed more than others before being buried.

Regarding the ontogenetic stage of the recovered specimens, the fact that it is very hard to associated elements to the same individual, makes is difficult to establish with certainty the number of juveniles, sub-adults and adults. However, most of the bones from *Caiuajara dobruskii* recovered are predominatly small, as can be exemplified by the humerus (Table S1 in File S1) and also the sizes of the skull (Figure 5). Therefore we can observe that most recovered specimens are predominantly juveniles or very young animals, with adults being quite rare, represented by only two skulls and three humeri.

It is also very difficult to establish precisely the wing span variation of the sample collected so far. Comparing the size of several postcranial elements with other tapejarids [1], [15], [16], particularly the humerus, allows us to estimate the wingspan variation of what is presently known of *Caiuajara dobruskii* between 0.65 and 2.35 m.

Most specimens were collected in two different levels less than 0.5 m apart vertically. A third level of accumulation with hundreds of bones of small individuals in a more restricted area is less than 0.5 m above the last one (Figure 1). A fourth one yielded only isolated elements, indicating distinct events that generated this pterosaur accumulation. Sedimentological data supports the interpretation that the Goio-Erê Formation was formed in a desert environment with interdunal wetland [9], [10]. So far there is no evidence of invertebrate or plant material.

The fact that several pterosaur individuals were found in such close association is compelling evidence that *Caiuajara dobruskii* was gregarious, as has been suggested based on similar evidences for other extinct reptiles, including dinosaurs [43], [44]. Previous evidences of this kind of social behavior were restricted to some specimens of *Quetzalcoatlus* sp. found in close proximity [45] and a concentration of the archaeopterodactyloid *Pterodaustro* in Argentina [6]. Besides those, close associations of pterosaur individuals are exceedingly rare, limited to fragmentary remains of unknown affinity from Chile [5], one duplicate bone in one nodule from the Romualdo Formation [46] and two pterosaur specimens from Kazakhstan [47], making the pterosaur bone bed in Cruzeiro do Oeste particularly important.

Based on the available information, we conclude that *Caiuajara dobruskii* lived in colonies around an inland lake situated in a desert. Although some parental care might have been possible, the fact that the postcranial skeleton does not differ among juveniles and adults suggests that the new species was precocial and most likely could fly at a very young age. Other researcher have also pointed out to this possibility (e.g., [48], [49]). The taphonomic and geological conditions suggest that individuals died around an oasis over the years, being exposed and gradually disarticulated. The degree of disarticulation was dependent on the exposure time. Episodic events (e.g., desert storms) likely carried the disarticulated and partially articulated skeletons to the bottom of the lake where they got eventually preserved. The presence of three main levels of accumulation in a section of less than one meter suggests that this region was home to pterosaur populations for an extended period of time. It is also plausible that *Caiuajara* was a migratory pterosaur that visited this area from times to time, although the first possibility is favored here. The causes of death remain unknown, although similarities with dinosaur drought-related mortality are striking [42]. However, it is also possible that desert storms could have been responsible for the occasional demise of these pterosaurs.

## Supporting Information

File S1 **Supporting information.** List S1, Specimens referred to *Caiuajara dobruskii* gen. et sp. nov. List S2, Minimum number of individuals of *Caiuajara dobruskii* gen. et sp. nov. List S3, Phylogenetic analysis, characters and character matrix. Table S1, Measurements of wing elements of *Caiuajara dobruskii* gen. et sp. nov. Table S2, Measurements of hindlimb elements and the pteroid of *Caiuajara dobruskii* gen. et sp. nov.
(DOC)
