# Supplementary material for: Discovery of a Rare Pterosaur Bone Bed in a Cretaceous Desert with Insights on Ontogeny and Behavior of Flying Reptiles
Source: PLoS One. 2014 Aug 13;9(8):e100005. doi: 10.1371/journal.pone.0100005 (PMC4131874; doi:10.1371/journal.pone.0100005)
Supplement: File S1 — Supporting information. List S1, Specimens referred to Caiuajara dobruskii gen. et sp. nov. List S2, Minimum number of individuals of Caiuajara dobruskii gen. et sp. nov. List S3, Phylogenetic analysis, characters and character matrix. Table S1, Measurements of wing elements of Caiuajara dobruskii gen. et sp. nov. Table S2, Measurements of hindlimb elements and the pteroid of Caiuajara dobruskii gen. et sp. nov. (DOC) [file pone.0100005.s001.doc]

Discovery of a rare pterosaur bone bed in a Cretaceous desert with insights on ontogeny and behavior of flying reptiles

**Supporting Information**

List S1) Specimens referred to *Caiuajara dobruskii* gen. et sp. nov.

CP.V 866 a.b - humerus, ulna, radius, coracoid and several vertebrae; CP.V 874 **-** incomplete skull; CP.V 875 - left jugal; CP.V 876 a.b, - skull and long bones; CP.V 878 - postcranial elements; CP.V 879 - postcranial elements; CP.V 978 **-** tibia; CP.V 980 - humerus; CP.V 982- humerus; CP.V 983 - ulna; CP.V 1000 - sternum; CP.V 1002 a.b **-** metacarpal, radius, ulna, humerus, and wing elements; CP.V 1007 **-** femur, right and left humerus, scapula, wing elements and other unidentified bones; CP.V 1008 **-** vervical vertebrae, humerus, radius, ulna, scapula, ribs, and wing elements; CP.V 1009 **-** metatarsals, humerus and unidentified elements; CP.V 1010 **-** right humerus, right femur, and unidentified elements; CP.V 1011 - humerus; CP.V 1012 - humerus; CP.V 1013 - humerus; CP.V 1014- humerus; CP.V 1015 - humerus; CP.V 1027 - skull; CP.V 1447 - skull and postcranial elements; CP.V 1448 - skull; CP.V 1451 - skull; CP.V 1452 - skull; CP.V 1884 - skull; CP.V 2029 - skull, femur and other postcranial elements.

Besides those, there are several hundreds of bones that can be referred to the new species which are housed in the CENPALEO and are being prepared.

List S2) Minimum number of individuals of *Caiuajara dobruskii* gen. et sp. nov.

This number is based on anterior portion of the skulls (rostrum), of the following specimens (in parentheses the number of individuals):

CP.V 865 (1), CP.V 867 (1), CP.V 868 (1), CP.V 872 (1), CP.V 873 (1), CP.V 874 (1), CP.V 875 (1), CP.V 876 (1),CP.V 999 (1), CP.V 1001 (1), CP.V 1003 (1), CP.V 1004 (1), CP.V 1005 (1), CP.V 1006 (1), CP.V 1023 (1), CP.V 1024 (1), CP.V 1027 (1), CP.V 1447 (1), CP.V 1448 (1), CP.V 1449 (1), CP.V 1450 (min. 14), CP.V 1451 (1), CP.V 1452 (1), CP.V 1884 (1), CP.V 2003 (1), CP.V 2029 (1), UEPG/DEGEO/MP-4151 (2), UEPG/DEGEO/MP-4152 (1), UEPG/DEGEO (no number). Besides those there is one unnumbered specimen in the UEPG/DGEO and 4 specimens with cranial elements being prepared at the Museu Nacional/UFRJ.

It should be noted that this number will increase as more specimens are being prepared.

List S3) Phylogenetic analysis, characters and character matrix.

In order to access the phylogenetic position *Caiuajara dobruskii* gen. et sp. nov., we performed a phylogenetic analysis using PAUP 4.0b10 for Microsoft Windows (Swofford, 2000) using the TBR heuristic searches performed using maximum parsimony. Characters were given equal weight and treated unordered (ACCTRAN setting). This analysis is based essentially on Vullo et al. [24]. The search conducted by PAUP including all three outgroups (*Ornithosuchus longidens*, *Herrerasaurus ischigualastensis* and *Scleromochlus taylori*) produced 9396 equally parsimonious trees with a length of 223 steps (consistency index = 0.6977; retention index = 0.8403; rescaled consistency index = 0.5954). If *Ornithosuchus* is deleted from the search, 3132 equally parsimonious trees with a length of 222 steps (consistency index = 0.7072; retention index = 0.8204; rescaled consistency index = 0.5802) are found. A strict consensus cladogram of this last search is shown in Figure 9.

CHARACTER LIST (per anatomical region)

SKULL

1. Dorsal margin of the skull:

0 - straight or curved downward

1 - concave

2 - only rostrum curved upward

2. Upper and lower jaw:

0 - laterally compressed

1 - comparatively broad

3. Rostral part of the skull anterior to the external nares:

0 - reduced

1 - elongated (less than half of skull length)

2 - extremely elongated (more than half of skull length)

4. Rostral end of premaxillae/maxillae downturned:

0 - absent

1 - present

5. Position of the external naris:

0 - above the premaxillary tooth row

1 - displaced posterior to the premaxillary tooth row

6. Process separating the external nares:

0 - broad

1 - narrow

7. External naris and antorbital fenestra:

0 - separated

1 - confluent forming a nasoantorbital fenestra

8. Naris and antorbital fenestra:

0 - shorter than 40% of the skull length

1 - longer than 40% of the skull length

9. Posterior margin of antorbital (or nasoantorbital) fenestra:

0 - straight

1 - concave

10. Nasoantorbital (or antorbital) fenestra extending dorsal to the orbit

0 - absent

1 - present

11. Shape of the orbit:

0 - subcircular

1 - circular

2 - circular, with open ventral margin

3 - piriform (elongated)

12. Orbit comparatively small and positioned very high in the skull:

0 - absent

1 - present

13. Position of the orbit relative to the nasoantorbital fenestra (naris + antorbital fenestra):

0 - same level or higher

1 - orbit lower than the dorsal rim of the nasoantorbital fenestra

14. Suborbital opening:

0 - absent

1 - present

15. Lower temporal fenestra:

0 - piriform, with ventral portion wider than dorsal

1 - piriform, with dorsal portion wider than ventral

2 - reduced (slit-like)

16. Premaxillary sagittal crest:

0 - absent

1 - present

17. Premaxillary sagittal crest, position:

0 - confined to the anterior portion of the skull

1 - starting anterior to the anterior margin of the nasoantorbital fenestra, extending beyond occipital region

2 - starting at about the anterior margin of the nasoantorbital fenestra, reaching the skull roof above the orbit but not extending over the occipital region

3 - starting close or at the anterior portion of the skull and extended over the occipital region

4 - starting at the posterior half of the nasoantorbital fenestra.

5 - starting at the middle portion of the nasoantorbital fenestra, extending above the occipital region.

18. Premaxillary sagittal crest shape:

0 - striated, low with a nearly straight dorsal margin

1 - striated, high, spike-like

2 - round dorsal margin, blade-shaped

3 - smooth, expanded anteriorly and forming a low rod-like extension posteriorly

4 - smooth, starting low anteriorly and very expanded posteriorly

19. Expansion on the anterior part of the premaxillary sagittal crest:

0 - absent

1 - present

20. Elongated dorsal premaxillary spike-like extension

0 - absent

1 - present

21. Expansion of the premaxillary tip:

0 - absent

1 - present, with premaxillary end high

2 - present, with premaxillary end dorsoventrally flattened.

22. Posterior ventral expansion of the maxilla:

0 - absent

1 - present

23. Maxilla-nasal contact

0 - broad

1 - absent

24. Free nasal process:

0 - absent

1 - present

25. Free nasal process position:

0 - placed laterally

1 - placed medially

26. Free nasal process size:

0 - long, almost reaching the ventral margin of the skull

1 - short

2 - knob-like (extremely reduced)

27. Free nasal process orientation:

0 - directed anteroventrally

1 - directed ventrally

28. Foramen on nasal process:

0 - absent

1 - present

29. Lacrimal extensively fenestrated

0 - absent

1 - present

30. Lacrimal process of the jugal thickness:

0 - broad

1 - thin

31. Lacrimal process of the jugal inclination:

0 - inclined anteriorly

1 - subvertical

2 - inclined posteriorly

32. Pronounced ridge on the lateral side of the jugal

0 - absent

1 - present

33. Anterior portion of the frontal rugose:

0 - absent

1 - present

34. Bony frontal crest:

0 - absent

1 - present

35. Bony frontal crest, position:

0 - confined to the posterior end of the skull

1 - starting above the orbit

2 - starting on the posterior half of the nasoantorbital fenestra

36. Bony frontal crest, shape:

0 - reduced and blunt

1 - short and spike-like, dorsally deflected

2 - narrow, directed posteriorly

3 - very high and broad, at least doubling the height of the skull above the orbit, directed posteriorly

4 - high, broad base and fan-shaped.

37. Bony parietal crest:

0 - absent

1 - present

38. Bony parietal crest shape:

0 - blunt

1 - constituting the base of the posterior portion of the cranial crest.

39. Posterior region of the skull rounded with the squamosal displaced ventrally:

0 - absent

1 - present

40. Position of the quadrate relative to the ventral margin of the skull:

0 - vertical or subvertical

1 - inclined about 1204 backwards

2 - inclined about 1504 backwards

41. Position of the articulation between skull and mandible:

0 - under the posterior half of the orbit or further

backwards

1 - under the middle part of the orbit

2 - under the anterior half of the orbit or further anterior

42. Helical jaw joint:

0 - absent

1 - present

43. Supraoccipital:

0 - does not extend backwards

1 - extends backwards

44. Foramen pneumaticum piercing the supraoccipital:

0 - absent

1 - present

45. Expanded distal ends of the paroccipital processes:

0 - absent

1 - present

46. Palatal ridge:

0 - absent

1 - discrete, tapering anteriorly

2 - strong, tapering anteriorly

3 - strong, confined to the posterior portion of the palate

47. Slight expansion of the palate close to the anterior opening of the nasoantorbital (or naris + antorbital) fenestra:

0 - absent

1 - present

48. Maxilla excluded from the internal naris:

0 - absent

1 - present

49. Opening between pterygoids and basisphenoid (interpterygoid opening):

0 - absent or very reduced

1 - present and larger than subtemporal fenestra

2 - present but smaller than subtemporal fenestra

50. Basisphenoid:

0 - short

1 - elongated

51. Mandibular symphysis:

0 - absent or very short

1 - present, at least 30% of mandible length

52. Step-like dorsal margin of the dentary in lateral view:

0 - absent

1 - present

53. Anterior tip of the dentary downturned:

0 - absent

1 - present

54. Dentary bony sagittal crest:

0 - absent

1 - present

55. Dentary bony sagittal crest, position:

0 - confined to the anterior third of the lower jaw

1 - extending close to the middle partion of the jaw

56. Dentary bony sagittal crest shape:

0 - small projection

1 - blade-like

2 - elongated ridge-like

3 - deep, broad in lateral view

4 - shallow

57. Position and presence of teeth:

0 - teeth present, evenly distributed along the jaws

1 - teeth absent from the anterior portion of the jaws

2 - teeth confined to the anterior part of the jaws

3 - jaws toothless

58. Largest maxillary teeth positioned posteriorly:

0 - absent

1 - present

59. Variation in the size of the anterior teeth with the 5th and 6th smaller than the 4th and 7th:

0 - absent

1 - present

60. Teeth with a broad and oval base:

0 - absent

1 - present

61. Teeth finely serrated:

0 - absent

1 - present

62. Peg-like teeth:

0 - absent

1 - present, 15 less on each side of the jaws

2 - present, more than 15 on each side of the jaws

63. Laterally compressed and triangular teeth:

0 - absent

1 - present

AXIAL SKELETON

64. Atlas and axis:

0 - unfused

1 - fused

65. Notarium:

0 - absent

1 - present

66. Postexapophyses on cervical vertebrae:

0 - absent

1 - present

67. Lateral pneumatic foramen on the centrum of the cervical vertebrae:

0 - absent

1 - present

68. Midcervical vertebrae:

0 - short, sub-equal in length

1 - elongated

2 - extremely elongated

69. Cervical ribs on midcervical vertebrae:

0 - present

1 - absent

70. Neural spines of the mid-cervical vertebrae, height:

0 - tall

1 - low

2 - extremely reduced or absent

71. Neural spines of the mid-cervical vertebrae, shape:

0 - blade-like

1 - spike-like

2 - ridge

72. Number of caudal vertebrae:

0 - more than 15

1 - 15 or less

73. Caudal vertebrae with elongated zygapophyses forming rod-like bony processes:

0 - absent

1 - present

74. Proximal caudal vertebrae with duplex centra:

0 - absent

1 - present

PECTORAL GIRDLE

75. Length of the scapula:

0 - subequal or longer than coracoid

1 - scapula shorter than coracoid (1 > sca/cor > 0.80)

2 - substantially shorter than coracoid (sca/cor < 0.80)

76. Proximal surface of scapula:

0 - elongated

1 - sub-oval

77. Shape of scapula:

0 - elongated

1 - stout, with constructed shaft

78. Coracoidal contact surface with sternum:

0 - flattened

1 - oval

79. Coracoidal contact surface with sternum:

0 - no developed articulation surface

1 - articulation straight or slightly concave

2 - articulation strongly concave

80. Posterior expansion on articulation surface of the coracoid with the sternum:

0 - absent

1 - present

81. Deep coracoidal flange:

0 - absent

1 - present

82. Broad tubercle on ventroposterior margin of coracoid:

0 - absent

1 - present

83. Cristospine:

0 - absent

1 - shallow and elongated

2 - deep and short

FORELIMB

84. Proportional length of the humerus relative to the metacarpal IV (hu/mcIV):

0 - hu/mcIV > 2.50

1 - 1.50 < hu/mcIV < 2.50

2 - 0.40 < hu/mcIV < 1.50

3 - hu/mcIV < 0.40

85. Proportional length of the humerus relative to the femur (hu/fe):

0 - hu/fe < 0.80

1 - 1.4 > hu/fe > 0.80

2 - hu/fe > 1.40

86. Proportional length of the humerus plus ulna relative to the femur plus tibia (hu+ul/fe+ti):

0 - humerus plus ulna about 0.80% or less of femur plus tibia length

(hu+ul/fe+ti < 0.80)

1 - humerus plus ulna larger than 0.80% of femur plus tibia length

(hu+ul/fe+ti > 0.80)

87. Pneumatic foramen on the ventral side of the proximal part of the humerus:

0 - absent

1 - present

88. Pneumatic foramen present on dorsal side of the proximal part of the humerus:

0 - absent

1 - present

89. Deltopectoral crest of the humerus:

0 - reduced, positioned close to the humerus shaft

1 - enlarged, proximally placed, with almost straight proximal margin

2 - enlarged, hatchet shaped, proximally placed

3 - enlarged, hatched shaped, positioned further down the humerus shaft

4 - enlarged, warped

5 - long, proximally placed, curving ventrally

90. Medial (= ulnar) crest of the humerus:

0 - absent or reduced

1 - present, directed posteriorly

2 - present, massive, with a developed proximal ridge

91. Distal end of the humerus:

0 - oval or D-shaped

1 - subtriangular

92. Proportional length of the ulna relative to the metacarpal IV (ul/mcIV):

0 - ulna 3.6 times longer than metacarpal IV (ul/mcIV > 3.6)

1 - length of ulna between 3.6 and two times the length of metacarpal IV (3.6 > ul/mcIV > 2)

2 - ulna between two times and the same length of metacarpal IV

(2 > ul/mcIV > 1)

3 - ulna about the same length or smaller than metacarpal IV (ul/mcIV < 1)

93. Diameter of radius and ulna:

0 - subequal

1 - diameter of the radius about half that of the ulna

2 - diameter of the radius less than half that of the ulna

94. Distal syncarpals, shape:

0 - irregular

1 - from a rectangular unit

2 - form a triangular unit

95. Pteroid:

0 - absent

1 - shorter than half the length of the ulna

2 - longer that half the length of the ulna

96. Metacarpals I - III:

0 - articulating with carpus

1 - metacarpal I articulates with carpus, metacarpals II and III reduced

2 - not articulating with carpus

97. Proportional length of the first phalanx of manual digit IV relative to the metacarpal IV (ph1d4/mcIV):

0 - both small and reduced

1 - both enlarged with ph1d4 over four times the length of mcIV (ph1d4/mcIV>4.0)

2 - both enlarged with ph1d4 about or less than two times the length of mcIV (ph1d4/mcIV<2.0) about 2 or smaller.

98. Proportional length of the first phalanx of manual digit IV relative to the tibiotarsus (ph1d4/ti):

0 - ph1d4 reduced

1 - ph1d4 elongated and less than twice the length of ti (ph1d4/ti smaller than 2.00)

2 - ph1d4 elongated about or longer than twice the length of ti (ph1d4/ti subequal/larger than 2.00)

99. Proportional length of the second phalanx of manual digit IV relative to the first phalanx of manual digit IV (ph2d4/ph1d4):

0 - both short or absent

1 - elongated with second phalanx about the same size or longer than first (ph2d4/ph1d4 larger than 1.00)

2 - elongated with second phalanx up to 30% shorter than first (ph2d4/ph1d4 between 0.70 - 1.00)

3 - elongated with second phalanx more than 30% shorter than first (ph2d4/ph1d4 smaller than 0.70)

100. Proportional length of the third phalanx of manual digit IV relative to the first phalanx of manual digit IV (ph3d4/ph1d4):

0 - both short or absent

1 - ph3d4 about the same length or larger than ph1d4

2 - ph3d4 shorter than ph1d4

101. Proportional length of the third phalanx of manual digit IV relative to the second phalanx of manual digit IV (ph3d4/ph2d4):

0 - both short or absent

1 - ph3d4 about the same size or longer than ph2d4

2 - ph3d4 shorter than ph2d4

102. Proportional length of the forth phalanx of manual digit IV relative to the first phalanx of manual digit IV (ph4d4/ph1d4):

0 - both short or absent

1 - both elongated, with the forth phalanx the longer than the first (ph4/d4/ph1d4>1.00)

2 - both elongated with the forth phalanx the same length or shorter, but longer than 35% the length of the first (1.00>ph4d4/ph1d4>0.35)

3 - both elongated with the forth phalanx less than 35% the length of the first (ph4d4/ph1d4<0.35)

HINDLIMB

103. Proportional length of the femur relative to the metacarpal IV (fe/mcIV):

0 - femur about twice or longer than metacarpal IV

(fe/mcIV > 2.00)

1 - femur longer but less than twice the length of

metacarpal IV (1.00 < fe/mcIV < 2.00)

2 - femur about the same length or shorter than

metacarpal IV (fe/mcIV < 1.00)

104. Length of metatarsal III:

0 - more than 30% of tibia length

1 - less than 30% of tibia length

105. Fifth pedal digit:

0 - with four phalanges

1 - with 2 phalanges

2 - with 1 or no phalanx (extremely reduced)

106. Last phalanx of pedal digit V:

0 - reduced or absent

1 - elongated, straight

2 - elongated, curved

3 - elongated, very curved (boomerang shape)

DATA MATRIX

***Ornithosuchus longidens***

0000000-00 000000---0 0000----00 0000--0-00 0000000000 0000--0000 0000000000 0000000000 0000000000 0000000000 000000

***Herrerasaurus ischigualastensis***

0000000-00 000000---0 0000----00 0000--0-00 0000000000 0000--0000 0000000000 0000000000 0000000000 0000000000 000020

***Scleromochlus taylori***

000?0?0-?0 ?000?0---0 0000----?? ???0--??00 0?????0??? ?0?0--00?0 ?0??0??00? ?000????00 ????00??0? ??0?0????? ?0????

***Anurognathus ammoni***

010001???0 ?????0---0 00?0----0? ?0?0--0-?? ??????0??? 0000--0000 110?0??0?? ?100??0??? ???011??10 000?1011?? ??001?

***Rhamphorhynchus muensteri***

0010100-00 000010---0 0000----00 0000--0-01 1000000110 1010--0000 1000000000 0010000010 0011110020 011?101222 221012

***Darwinopterus modularis***

0010101000 0000112000 0011010?00 1000--0-01 1?0?0?0??? 0000--0000 120?000100 00?0?????? ???1?????? ?1????21?? ??????

***Pterodactylus antiquus***

0010101000 000010---0 0011001000 1000--0-12 1?0?000??? 1000--0000 1200000111 0100000010 0012110050 021?102122 222020

***Nyctosaurus gracilis***

0010101000 0000?0---0 0010----00 1000--0-0? 110?000121 1000--3000 1001110010 0100000010 0013111030 ?312222222 2?20??

***Nemicolopterus crypticus***

0010101000 0000?0---0 0011110001 1000--0-0? 2?????0??? 1000--3000 100?0?001? ?100000??? 0???11?15? ?2????21?2 2?2120

***Pteranodon longiceps***

1020101010 300010---0 001112-000 1101221101 2101000121 1000--3000 1001111010 1101110120 0012111041 1312222222 232020

***Istiodactylus latidens***

0010101100 2???10---0 0?11110?01 21?0--0-01 2?01?00??1 0000--2000 101?1110?0 1???111020 002?1?0141 1?22?????? ??????

***Nurhachius ignaciobritoi***

0010101100 ?000?0---0 001?????01 110??????1 2?????0??? ?000--2000 10101??010 1???1?1020 0?22110?41 ?222?1212? ??21??

***Tropeognathus mesembrinus***

0010101000 3000110200 101?????00 1101001001 2101020121 1001010000 100??????? ?????????? ?????????? ?????????? ??????

***Anhanguera santanae***

0010101010 3000110200 1011100100 1101001001 2101010121 100??10010 1001011010 11??211021 00????0141 1?22?1???? ??????

***Anhanguera blittersdorffi***

0010101010 3000110200 101?????00 1101001001 2101010121 1001010010 100??????? ?????????? ?????????? ?????????? ??????

***Anhanguera piscator***

0010101010 3000110200 1011100100 1101001001 21010?0121 1001010010 1001011010 1101211121 0022110141 1222?1???? ??2120

***Ludodactylus sibbicki***

0010101000 000010---0 ?011100100 01011?1101 110?0?0??? 1000--0010 100??????? ?????????? ?????????? ?????????? ??????

***Dsungaripterus weii***

2010101000 1101111100 0110----00 1001211101 2111110121 1000--1101 1001111010 010?000??? 0?2210??5? 0311?12122 2?2120

***"Phobetor" parvus***

0010101000 1101111100 0110----00 1001211101 2?1?1?0??? 1000--1101 100??????0 ?????????? ?????????? ???1?????? ??????

***Quetzalcoatlus sp.***

0010101000 3010?14?00 0010----00 100??????1 21???001?? 1000--3000 1001110212 2?0?000020 10?20?1052 0311??2132 2?2?20

***Azhdarcho lancicollis***

?????????? 3????????? ?????????? ?0???????? ?????????? ??????3000 10?1110212 2????????? ??????105? 0????????? ??????

***Zhejiangopterus linhaiensis***

0010101000 3010?0---0 0010----00 1000--0-01 2?1???0??? ?000--3000 10011??212 2?0?000??? 10?200??5? ?31?2?213? ??2???

***Chaoyangopterus zhangi***

1010101??? ?0???????0 00???????? ?????????? ??????0??? 1000--3000 100?010110 0???000??? 00?200???? ?31?2?2122 2321?0

***Shenzhoupterus chaoyangensis***

1010101101 301020---0 001?????0? 2001241101 2?1???0??? 1000--3000 100?01011? ????0??0?? 0??200???? ?31???2122 2321??

***Tupuxuara leonardii***

0010101100 3010213400 001?????01 1001231101 2111130121 1001123000 1001111010 0???000020 0122101052 0311?1213? ??2???

***Thalassodromeus sethi***

0010101100 3010213400 001112-001 1001231101 2111130121 1000--3000 100??????? ?????????? ?????????? ?????????? ??????

***Tupandactylus imperator***

0011101100 3010213301 001??????1 1001221101 2?1??????? ???1133000 100??????? ?????????? ?????????? ?????????? ??????

***Europejara olcadesorum***

?01??????? ????2????? ?01??????? ????????01 20?????1?1 1101133000 100??????? ?????????? ?????????? ?????????? ??????

***Caiuajara dobruskii*** **nov gen., nov sp.**

00111011?? 3010213301 00???????1 0????????? 20???01??? 1101133000 000?0110?0 0?0?00002? 01?21?1152 03112????? ??????

***Tapejara wellnhoferi***

0011101100 3010213300 0011111011 1001221101 2011101121 1101133000 100?011010 0?0?000020 01?2101152 03112?21?? ??2120

***Sinopterus dongi***

0011101100 3010?13300 0011100011 1011211101 2?1???1??? ?101143000 100?010110 0???000020 0??2111?52 ?31?2?2122 2321??

***Eopteranodon* *lii***

001110110? ?????13300 00???????1 1????????? ?1???0???? 1?01143000 000??1???? 0?????0??? ???211??5? ?31?2?2122 2?2???

**"*Huaxiapterus*" *corollatus***

0011101??? ?????1?310 001??????? ?????????? ?????????? ?101143000 100?01011? ????000??? 0??211??5? ?3????2132 2321??

**"*Huaxiapterus*" *benxiensis***

0011101100 ?0???13310 001??????1 10?1211101 2?1???1??? 1101143000 100?01011? ?????????? ???2?????? ?3??2?2122 2?2120

Table S1) Measurements of wing elements of *Caiuajara dobruskii* gen. et sp. nov.

| Bones / Specimens | hu | ul | mcIV | ph1d4 | ph2d4 | ph3d4 | ph4d4 |
| --- | --- | --- | --- | --- | --- | --- | --- |
| CP.V 870 | ~ 41.0(r) |  |  |  |  |  |  |
| CP.V 866a | 38.8 (r) | 49.5 |  |  |  |  |  |
| CP.V 869 | ~39.0(l) | 50.4 |  |  |  |  |  |
| CP.V 872a | 83.9(r) | 109.8 |  |  | 121.4 |  |  |
| CP.V 1001a | 91.3(r) |  | 124,5(r)  109.4 |  |  |  |  |
| CP.V 1001b | 79.2(r)  79.2(l) | 113.7 | 110,3(l) |  |  | 104.5 |  |
| CP.V 1002 | 77.0(l) | 99.3 |  |  |  |  |  |
| CP.V 1006 | 90.6(r)  82.7(r) |  |  | 170.0 | 166.6  163.2 | 162.4 | 49.4 |
| CP.V 1007 | 65.3(r) | ~90.0 |  |  |  |  |  |
| CP.V 1009 | 72.9(l) |  | 129,4(l) |  |  |  |  |
| CP.V 1010 | 72.4(r) |  |  |  |  |  |  |
| CP.V 1013 | 113.6(l) |  |  |  |  |  |  |
| CP.V 1014 | 115.4(r) |  |  |  |  |  |  |
| CP.V 1015 | 115.6(l) |  |  |  |  |  |  |
| CP.V 1023 |  |  |  | 165.0 | 137.7 |  |  |
| CP.V 1024 | 74.1 (l) |  |  |  |  |  |  |
| CP.V 1450 | 29.0(r)  31.0(-)  32.0(r)  36.0(l)  41.0(r)  42.0(r)  43.0(r)  51.0(l) |  | 51.0  54.0  60.0  68.0 | 52.0  60.0  68.0 |  |  |  |

­­

Table S2) Measurements of hindlimb elements and the pteroid of *Caiuajara dobruskii* gen. et sp. nov.

| Bones / Specimens | fe | ti | mt4 | pt |
| --- | --- | --- | --- | --- |
| CP.V 869 | 29 |  |  |  |
| CP.V 872 a | 84.1 ( r) | 103.0 |  |  |
| CP.V 1002 |  | 95.0 |  |  |
| CP.V 1006 |  |  |  | 40.7 |
| CP.V 1007 | 74.5 |  |  |  |
| CP.V 1010 | 82.7( r) |  |  |  |
| CP.V 1023 | 69.3 |  |  |  |
| CP.V 1024 |  |  | 56.5 |  |
| CP.V 1025 | 118.6(l) |  |  |  |
| CP.V 2029 | 114.0(r) |  |  |  |

Abbreviations: hu - humerus, fe - femur, mcIV - metacarpal IV, mt4 - metatarsal 4, ph1d4 - first phalanx of manual digit IV, ph2d4 - second phalanx of manual digit IV, ph3d4 - third phalanx of manual digit IV, ph4d4 - forth phalanx of manual digit IV, pt - pteroid, ti - tibia, ul - ulna, r - right, l - left.
